# Cytomegalovirus infection disrupts the influence of short-chain fatty acid producers on Treg/Th17 balance

**DOI:** 10.1186/s40168-022-01355-3

**Published:** 2022-10-10

**Authors:** Ning Chin, Nicole R. Narayan, Gema Méndez-Lagares, Amir Ardeshir, W. L. William Chang, Jesse D. Deere, Justin H. Fontaine, Connie Chen, Hung T. Kieu, Wenze Lu, Peter A. Barry, Ellen E. Sparger, Dennis J. Hartigan-O’Connor

**Affiliations:** 1 California National Primate Research Center, University of California, Davis, Davis, USA; 2 Department of Medical Microbiology and Immunology, School of Medicine, University of California, Davis, Davis, USA; 3 Center for Immunology and Infectious Diseases, University of California, Davis, Davis, USA; 4 Department of Medicine and Epidemiology, School of Veterinary Medicine, University of California, Davis, Davis, USA; 5 Division of Experimental Medicine, Department of Medicine, University of California, San Francisco, San Francisco, USA

**Keywords:** Host-microbe interactions, Microbiome, Cytomegalovirus infection, Immunophenotype, Elastic net, Rhesus macaque, 16S analysis

## Abstract

**Background:**

Both the gut microbiota and chronic viral infections have profound effects on host immunity, but interactions between these influences have been only superficially explored. Cytomegalovirus (CMV), for example, infects approximately 80% of people globally and drives significant changes in immune cells. Similarly, certain gut-resident bacteria affect T-cell development in mice and nonhuman primates. It is unknown if changes imposed by CMV on the intestinal microbiome contribute to immunologic effects of the infection.

**Results:**

We show that rhesus cytomegalovirus (RhCMV) infection is associated with specific differences in gut microbiota composition, including decreased abundance of Firmicutes, and that the extent of microbial change was associated with immunologic changes including the proliferation, differentiation, and cytokine production of CD8^+^ T cells. Furthermore, RhCMV infection disrupted the relationship between short-chain fatty acid producers and Treg/Th17 balance observed in seronegative animals, showing that some immunologic effects of CMV are due to disruption of previously existing host-microbe relationships.

**Conclusions:**

Gut microbes have an important influence on health and disease. Diet is known to shape the microbiota, but the influence of concomitant chronic viral infections is unclear. We found that CMV influences gut microbiota composition to an extent that is correlated with immunologic changes in the host. Additionally, pre-existing correlations between immunophenotypes and gut microbes can be subverted by CMV infection. Immunologic effects of CMV infection on the host may therefore be mediated by two different mechanisms involving gut microbiota.

Video Abstract

**Supplementary Information:**

The online version contains supplementary material available at 10.1186/s40168-022-01355-3.

## Background

Cytomegalovirus (CMV) is a β-herpesvirus that infects an estimated 80% of the global population [1] and drives significant changes in immune-cell phenotypes and functions [2]. Despite an arsenal of host immune responses against CMV, the infection is never cleared; instead, the virus establishes latency and can reactivate and cause disease when the host is immunocompromised, such as in cases of congenital infection, organ transplants, or acquired immunodeficiency syndrome (AIDS) [3]. Throughout the course of asymptomatic and predominantly latent infection, periodic viral reactivation can occur and stimulate the immune system, resulting in up to 10% of both CD4^+^ and CD8^+^ memory T-cell populations having specificity for CMV [4]. A large effect of persistent viral infection on the adaptive immune compartments might be expected, but surprisingly, CMV has equally substantial, durable effects on innate immune cells. Despite relative infrequency of CMV infection among antigen-presenting cells (APC), for example, the phenotype of these cells is transformed after infection [5]. Similarly, natural killer (NK) cells are impacted, and the imposed changes appear lifelong [6]. The virus’s natural ability to promote large expansions of adaptive immune cells prompted investigations into using rhesus CMV (RhCMV) as a vaccine vector to protect against the simian immunodeficiency virus (SIV), a model for human immunodeficiency virus (HIV) [7, 8]. We showed that vaccination with RhCMV-vectored vaccines is associated with IL-15-dependent expansion of innate-memory cells with SIV killing function [9].

Other environmental factors such as the gut microbiome have been shown to interact with the host to modify cytokine production capacity [10], potentially modifying vaccine responses. We demonstrated that infant diet and associated gut microbiota differences significantly impact immune development over the first 3–5 years of life [11, 12]. Additionally, type-1 diabetes onset in genetically predisposed infants was associated with decreased alpha diversity of the gut microbiome [13]. Inoculation of germ-free mice with segmented filamentous bacteria was shown to be critical for the development of Th17 cells [14], which are important for maintenance of the intestinal barrier and robust mucosal immunity [15]. Given the importance of microbes in shaping the host immune system, it is not surprising that certain constituents of the gut microbiota, including members of the phyla Actinobacteria and Firmicutes, have been associated with better vaccine responses, while Proteobacteria and Bacteroidetes have been associated with poor responses [16].

Interactions between viral infections and the commensal microbiota further complicate their relationships with host immunity. The gut microbiota can promote viral infection in some contexts but suppress viral infection in others [17]. For example, short-chain fatty acids (SCFAs) produced by commensal bacteria have been shown to reactivate latent herpesviruses such as Epstein-Barr virus and Kaposi’s sarcoma-associated herpesvirus [18], but lactic acid and *Lactobacillus* cell-wall components have been shown to inhibit herpes simplex virus activity [19, 20]. The persistence of murine norovirus differs per gut microbiota composition [21]. Another mouse study found that previous infection with murine CMV altered responses to yellow fever vaccination [22]. Moreover, mice latently infected with murine CMV exhibited greater resistance to sublethal doses of *Listeria monocytogenes* and reduced *Yersinia pestis* replication and spread [23]. Subclinical infections with RhCMV in adult macaques have been shown to induce changes in the gut microbiota and result in reduced immune responses to influenza A vaccination [24]. Since both CMV and the gut microbiota induce signaling cascades that control immune responses, it is reasonable that the affected pathways may overlap and interact [25, 26].

Nonetheless, interactions between the gut microbiota and chronic viral infections have only been superficially explored. Rhesus macaques represent a particularly useful model for studying how chronic viral infections alter relationships between gut microbiota and host [27]. Rhesus immune cells and many features of the adaptive response are similar to those found in humans [28]. Studies of RhCMV have demonstrated effects on host immunity similar to those apparent after human CMV (HCMV) infection [29]. The pathogenesis of fetal infections with RhCMV and HCMV are similar, making RhCMV an ideal model for study of HCMV pathogenesis, vaccines, and effects on the immune system [30]. To investigate the impact of RhCMV on gut microbe-host relationships, we studied the gut microbiotas and immune systems of RhCMV-seropositive and -seronegative infant macaques. We found that RhCMV infection had a direct effect on abundance of certain bacterial taxa in the gut and altered relationships between gut microbial taxa and immune-cell subsets.

## Methods

### Study design

Rectal swabs and blood samples were collected from 5- to 11-month-old infant rhesus macaques that were seropositive (RhCMV+, *n* = 29) or seronegative (RhCMV−, *n* = 38) for RhCMV. Animals were co-housed in outdoor corrals; samples were collected from all available animals in the correct age range at the time of sampling, without selection. Rectal swabs were stored in RNAlater™ Storage Solution (Sigma-Aldrich) at −70 °C until DNA extraction. Peripheral blood mononuclear cells (PBMCs) were isolated by gradient density purification using Lymphocyte Separation Medium (MP Biomedicals, LLC), and then washed in medium containing fetal bovine serum and 10% dimethyl sulfoxide (DMSO) before cryopreservation in liquid nitrogen prior to analysis. Stool samples were also collected from a separate longitudinal study in which 24 adult female indoor-housed RhCMV-negative rhesus macaques were vaccinated with a RhCMV 68-1-based vaccine vector [31]. Baseline samples were collected 2 weeks prior to vaccination, and post-vaccination samples were collected 3 weeks post vaccination.

### DNA extraction and 16S rDNA sequencing

DNA from rectal swabs and stools was extracted using the MoBio PowerSoil kit (Qiagen). Amplicon libraries were generated by amplifying the V3-V4 or V4 variable region of 16S rRNA genes using primers 319F and 806R or 515F and 806R, respectively [32]. Both forward and reverse primers contained a unique 8-nt barcode, a primer pad, a linker sequence, and the Illumina adaptor sequences. Each sample was barcoded with a unique forward and reverse barcode combination. PCR reactions contained 1 unit KAPA2G Robust HotStart Polymerase (Kapa Biosystems), 1.5 mM MgCl_2_, 10 μmol of each primer, 10 mM dNTPs, and 1 ul of DNA. PCR conditions were as follows: an initial incubation at 95 °C for 2 min; 30 cycles of 95 °C for 15 s, 50 °C for 20 s; 72 °C for 20 s, and a final extension at 72°C for 3 min. The final product was quantified on the Qubit instrument using the Qubit High Sensitivity DNA kit (Invitrogen), and individual amplicon libraries were pooled, cleaned by AMPure XP beads (Beckman Coulter), and sequenced using a 250 bp paired-end method on an Illumina MiSeq instrument in the Genome Center DNA Technologies Core, University of California, Davis. The quality of sequencing reads was checked using FastQC. Sequences were trimmed and annotated to the genus level using the DADA2 package [33] to Greengenes database version 13_8 [34] within the R 4.1.1 software [35] using RStudio [36]. The numbers of reads per sample per bacterial feature were stored in a matrix and used in downstream statistical analysis.

### Immune-cell phenotyping by flow cytometry

Immune cells were stained and analyzed as described in previously published paper using samples from the same animal cohort [9]. Distribution of innate cells and T-cell subsets in peripheral blood samples and their activation status were determined by flow cytometry using freshly isolated PBMC samples. The following antibodies were used: anti-CD3-Alexa 700, anti-CD95-APC (clone DX2), anti-CD28-APC-H7 (clone CD28.2), anti-CD8–PE-Cy5.5 (clone 3B5), anti-CD4–BV650 (clone L200), anti-HLADR-ECD (clone Immu-357), anti-CD14-Qdot 605 (clone TüK4), anti-CD16-PacBlue (clone 3G8), anti-CD20-ECD (clone B9E9), anti-CD11c-AF700 (clone 3.9), anti-CD123-PerCP-Cy™5.5 (clone 7G3), anti-CD80-FITC (clone L307.4), anti-CD83-PE (clone HB15e), and anti-CD86-APC (clone FUN-1). A cell viability dye (Invitrogen Aqua LIVE/DEAD Fixable Dead Cell Stain) was included to discriminate live from dead cells. Cells were washed and permeabilized using a Fix/Perm kit (BioLegend) according to the manufacturer’s instructions, intracellularly stained with anti-Ki67–Alexa 488 (clone B56) and fixed in phosphate-buffered saline containing 1% paraformaldehyde. Data were acquired on Aria or Fortessa cytometers (BD Biosciences) and analyzed using FlowJo software version 10.3 (BD Life Sciences).

### Intracellular cytokine immunostaining

Immune cells were stained and analyzed as described in previously published paper using samples from the same animal cohort [9]. To measure the level of cytokine production in response to mitogenic stimulation, PBMC (1 million cells) were incubated for 4 h at 37 °C with phorbol 12-myristate 13-acetate (50 ng/ml) and ionomycin (1 μg/ml) in complete RPMI 1640 medium and with GolgiPlug (5 μg/ml). Cells were washed and immunostained with anti-CD3-PacBlue (clone SP34-2), anti-CD8–PE-Cy5.5 (clone 3B5), anti-CD4–BV650 (clone L200), anti-CD95-APC (clone DX2), and anti-CD28-APC-H7 (clone CD28.2) and a stain reagent (Invitrogen Aqua LIVE/DEAD Fixable Dead Cell Stain) to exclude dead cells. Cells were washed and permeabilized using a Cytofix/Cytoperm kit (BD Biosciences) according to the manufacturer’s instructions and intracellularly immunostained with anti-IL-17–PE (clone eBio64DEC17), anti–IFN-γ-PE-Cy7 (clone B27), and anti-TNFα-Alexa Fluor 700 (clone Mab11). Cells were finally washed and fixed in phosphate-buffered saline containing 1% paraformaldehyde. Data were acquired on Aria or Fortessa cytometers (BD Biosciences) and analyzed using FlowJo software version 10.3 (BD Life Sciences).

### Statistical analysis

R-4.1.1 in RStudio was used for all statistical analysis [35, 36]. R packages phyloseq [37], vegan [38], limma [39], glmnet [40], and pROC [41] were used to filter taxa with < 5% prevalence, calculate distance matrices, perform differential abundance analysis, perform elastic-net logistic regression, and assess robustness of elastic-net algorithm, respectively. When assessing relationships between bacterial taxa and immune parameters (Table 2), residuals of each model were checked for homoscedasticity (Breusch-Pagan test) and normal distribution (Shapiro-Wilk test). *P*-values were adjusted for multiple testing using false discovery rate estimation with the qvalue package [42]. All the data were analyzed, without elimination of any outliers.

## Results

### Gut microbial communities of RhCMV+ and RhCMV− animals are similar at the phylum level but cluster separately at the genus level

**Fig. 1:**
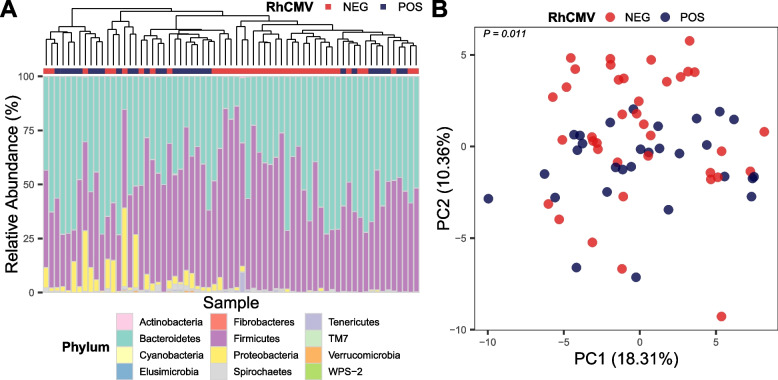
Gut microbial communities of RhCMV+ and RhCMV− animals are similar at the phylum level but cluster separately at the genus level. **A** Relative abundance of microbiota for each animal at the phylum level. Samples are ordered by complete-linkage clustering based on Aitchison distance. Animals are color coded as RhCMV− (red) and RhCMV+ (blue) on top of the bar graph. **B** PCA plot of all samples analyzed at the genus level, based on Aitchison distance

To test the impact of RhCMV infection on gut microbiota, we performed 16S ribosomal RNA gene sequencing on rectal swabs from 67 macaques aged 5 to 11 months, housed outdoors, and screened for anti-RhCMV antibodies. 29 seropositive animals and 38 seronegative animals were sampled (Table 1). An average of 81,000 raw reads were acquired per sample; an average of 65% ± 8% were retained after processing with the DADA2 pipeline [33], resulting in a mean of 52,000 analyzed reads per sample. We used Greengenes database version 13_8 [34] for taxonomic assignment. Bacterial sequence counts were agglomerated to the genus level, and reads not classified to the genus level were assigned to the lowest taxonomic assignment. Taxa that were not present in at least 5% of the samples were filtered, resulting in 92 bacterial taxa for downstream analysis. Bacteroidetes and Firmicutes were the most abundant phyla in all samples, accounting for 49% and 47% of reads, respectively (Fig. 1A). Alpha diversities within RhCMV+ and RhCMV− animals were assessed using Shannon’s diversity index, evenness, and richness, and no significant differences were seen (Wilcoxon rank-sum test *P* > 0.05). Beta diversity at the genus level was assessed using complete-linking clustering and principal component analysis (PCA) with Aitchison distances. In this analysis, animals were shown to cluster by RhCMV serostatus (Fig. 1B, *P* = 0.01).

**Table 1:** Study groups

Group	No. of animals	Mean ± SD (range)		Sex (male:female)	Housing
		Age (months)	Weight (kg)		
RhCMV seronegative	38	8.5 ± 1.2 (5.7–11.1)	1.8 ± 0.3 (1.3–2.4)	22:16	Outdoor
RhCMV seropositive	29	8.6 ± 0.9 (6–10.8)	1.8 ± 0.2 (1.4–2.2)	18:11	
RhCMV seronegative, RhCMVvectored vaccine recipient (strain 68-1)	24	51.3 ± 13.1 (39–87)	6.1 ± 1.8 (4.0–11.4)	0:24	Indoor

### RhCMV-seropositive animals and those experimentally infected with RhCMV68-1 vaccine vector had significantly decreased abundances of bacteria from phylum Firmicutes

**Fig. 2:**
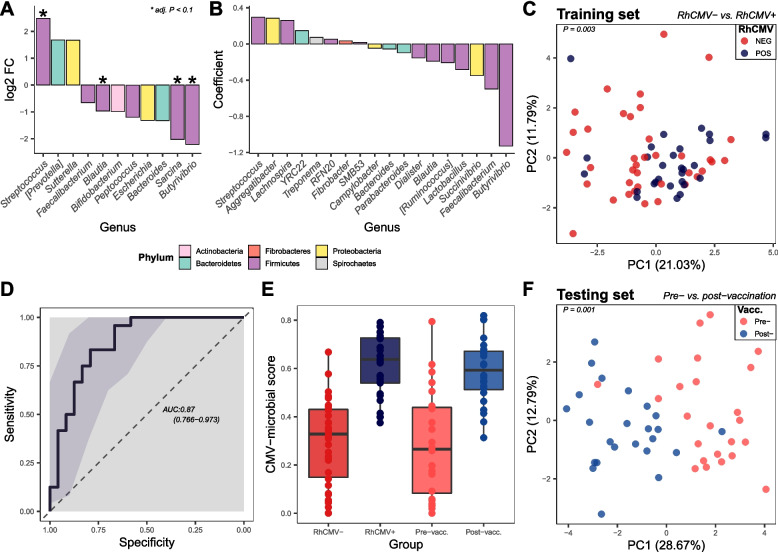
RhCMV-infected animals had significantly decreased abundance of bacteria from the order Clostridia. **A** Differentially abundant bacterial genera analyzed using the limma-voom pipeline with *P* < 0.05 (*adjusted *P* < 0.1). **B** Features selected by elastic-net regression to differentiate between RhCMV− and RhCMV+ animals. **C** PCA plot generated using log-transformed counts of genera selected by elastic net within the training set. **D** ROC curve to assess the robustness of elastic-net output. **E** Summary score for each animal generated by the elastic net. **F** PCA plot generated using log-transformed counts of genera selected by elastic-net within the testing set (RhCMV-vectored vaccine recipients)

To detect specific differences in microbial communities associated with RhCMV infection, we analyzed genus-level data (86% of total reads) using linear modeling of abundances with variances moderated by an empirical Bayes procedure (limma-voom, ref. [39, 43]). We found that 11 of 62 detected bacterial genera, six of which were Firmicutes, had different abundance in animals infected with RhCMV (Fig. 2A, unadjusted *P* < 0.05). Four genera were determined to be significantly changed (adjusted *P* < 0.1) after adjustment for multiple comparisons. *Butyrivibrio*, *Sarcina*, and *Blautia*, all known short-chain fatty acid (SCFA) producers [44–46], were less abundant in RhCMV+ animals; *Streptococcus* was more abundant.

We used elastic-net logistic regression to attempt better modeling of the RhCMV-associated microbiota despite multicollinearity in the dataset [47, 48]. A 10× cross-validation was performed on a grid of alpha values to determine the optimal alpha (mixture between ridge and lasso) and lambda. Eighteen bacterial genera were found to contribute to an optimal ensemble associated with RhCMV infection (Fig. 2B). *Streptococcus*, *Aggregatibacter*, *Lachnospira*, *YRC22*, *Treponema*, *RFN20*, *Fibrobacter*, and *SMB53* had positive coefficients, signifying that they have positive correlations with RhCMV infection, while *Campylobacter*, *Bacteroides*, *Parabacteroides*, *Dialister*, *Blautia*, *Ruminococcus*, *Lactobacillus*, *Succinivibrio*, *Faecalibacterium*, and *Butyrivibrio* had negative coefficients. As expected, when abundances of these 18 genera were reduced to two principal components, plotting demonstrated substantial separation of the RhCMV-seronegative vs. -seropositive animals (Fig. 2C). To determine if RhCMV infection itself was the cause of such associations, we performed 16S rRNA sequencing of stool samples taken 2 weeks before vs. 3 weeks after administration of an RhCMV68-1 vaccine vector [7]. The elastic-net model that had been trained using samples from animals with natural infection (“training set,” above) was applied to the cohort of vaccinated animals (“testing set”) to determine if the 18 selected genera were good identifiers of previous RhCMV exposure via vaccination. The area under the receiver operating characteristic (ROC) curve was 0.87 with a confidence interval of 0.766–0.973, showing that the method was robust in differentiating RhCMV naïve vs. RhCMV-infected or -vaccinated animals (Fig. 2D). The application of elastic net resulted in a summary score calculated by the model to describe the degree of “RhCMVness” in the microbiota of individual animals (Fig. 2E), which showed clear separation between RhCMV− vs. RhCMV+ and pre- vs. post-vaccinated animals. The results were further confirmed by examination of a principal-component plot of the log2-transformed counts of important taxa as determined by elastic-net regression, in which pre- and post-vaccinated animals (“testing set,” Fig. 2F; PERMANOVA *P* = 0.001) clustered separately from each other.

### Bacterial features were correlated with immune phenotypes

**Fig. 3:**
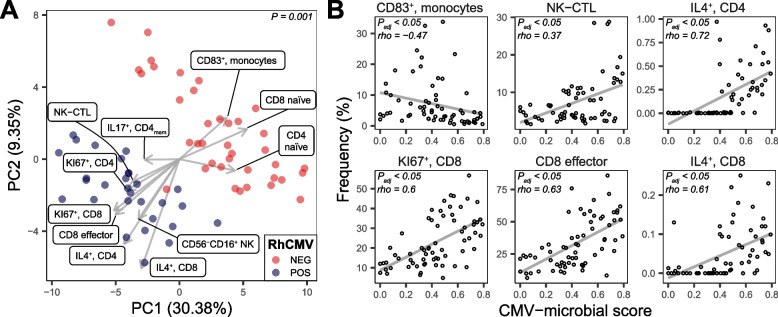
Immune cell types correlated with microbiome constituents. **A** PCA plot of immune markers. Loadings of important differences associated with RhCMV infection are shown. **B** Immune cell subsets correlated with the CMV-microbe score shown in Fig. 2E

We described immunologic changes resulting from CMV infection in a previously published paper whose experiments were performed in parallel with the microbiome investigation [9]. Animals were seen to cluster according to RhCMV serostatus on a PCA plot summarizing immune-cell frequencies, demonstrating the large and consistent immunologic change imposed by this infection (Fig. 3A, PERMANOVA, *P* < 0.05; see ref. [2]). Specific differences in immune-cell populations between RhCMV+ and RhCMV− animals were consistent with those previously reported, including lower frequency of naïve and greater frequency of memory/effector CD4^+^ and CD8^+^ T cells (Fig. 3A; ref [23]). Proliferation of T cells, marked by Ki-67 expression, was also highly correlated with RhCMV infection (Fig. 3A).

To test for a possible association between immunologic and microbial changes, we used the CMV-microbe score from Fig. 2E as a summary score for microbial change and then searched for associations between these scores and circulating immune cells. Frequencies of 48 out of 84 (57%) immune subsets examined were significantly associated with the CMV-microbe score (Spearman rank test, adjusted *P* < 0.05; Table S1 and examples in Fig. 3B), suggesting an important relationship between CMV serostatus, the gut microbiota, and immune markers.

### Cytomegalovirus subverts relationships between gut bacteria and immunophenotypes

**Fig. 4:**
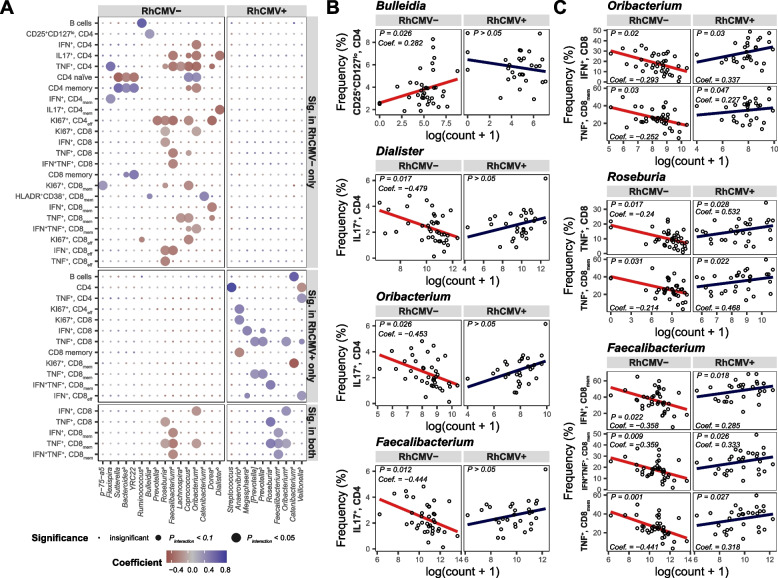
RhCMV subverts relationships between immune-cell subsets and gut bacteria. **A** Significant associations between immune cells and gut bacterial abundance that were changed by RhCMV infection, as indicated by a significant interaction term in regression. The significance of interaction term was indicated by different sizes (largest have *P* < 0.05, medium have 0.05 ≤ *P* < 0.1, smallest have *P* ≥ 0.1). Top panels show significant bacterial-immune correlations within RhCMV− animals only; middle panels show significant bacterial-immune correlations within RhCMV+ animals only; bottom panels show significant bacterial-immune correlations in both RhCMV+ and RhCMV− animals. Red signifies negative correlations, while blue signifies positive correlations. ^a^Gram-positive and ^b^gram-negative bacterial genera known to produce SCFA. **B** Significant correlations between Treg (CD25^+^CD127^lo^ CD4^+^ T cells) or Th17 (IL17^+^ CD4^+^ T cells) and known SCFA producers *Bulleidia*, *Dialister*, *Oribacterium*, and *Faecalibacterium* in RhCMV− animals but not RhCMV+ animals, also shown in the top panel of Fig. 4A. **C** Significant immune correlations detected in both RhCMV− and RhCMV+ animals with *Oribacterium*, *Roseburia*, and *Faecalibacterium*, also shown in the bottom panel of Fig. 4A

We hypothesized that the immunologic impact of RhCMV infection would be strong enough to swamp many preexisting effects of commensal gut bacteria on immunity, subverting existing gut microbiota-host relationships. We searched for features of the host immune system whose relationship to specific bacterial taxa was modified by RhCMV infection (indicated by a significant interaction term in a multivariate analysis accounting for age). To avoid spurious significance due to sparse read counts, we further filtered taxa not represented in at least 50% of samples, resulting in 2905 total comparisons between 35 bacterial genera and 83 immune markers. Sixty-seven comparisons were statistically significant for both the overall *F*-test (*P* < 0.05) and the interaction term (*P* < 0.05) while also passing basic model checks (Table 2; see “Methods”). Most of the significantly altered relationships detected (44 of 63) reflected significant correlations in the RhCMV-seronegative animals that were not observed in the seropositive cohort, suggesting destruction of a preexisting relationship by RhCMV, confirming our hypothesis (Table 2, marked “RhCMV− only” at right, and examples in Fig. 4A, top section). Many such cases involved CD4^+^ and CD8^+^ T-cell populations known to be highly impacted by RhCMV. Specifically, among RhCMV-seronegative animals, cytokine-producing T-cell subsets were negatively correlated with genera from the Lachnospiraceae family (*Roseburia*, *Oribacterium*, *Coprococcus*, *Lachnospira*, and *Dorea*), often short-chain fatty acid producers [44, 45], but these relationships were diminished in seropositive animals (Table 2 and examples in Fig. 4A, top section). Additionally, in seronegative macaques, SCFA producers were associated with Th17/Treg balance through positive correlations with Tregs (marked “CD25^+^, CD127^lo^, CD4”) and negative correlations with Th17 cells (“IL17^+^, CD4”; Fig. 4A, top section, and Fig. 4B). In rarer cases, relationships not seen in seronegative macaques were present in seropositive macaques (16 of 67 interactions; Fig. 4A, middle section). Genera from the Veillonellaceae family (*Veillonella*, *Megasphaera*, and *Anaerovibrio*) were seen to have more impact on the immune system in seropositive macaques (Table 2 and examples in Fig. 4A, middle section), suggesting that in CMV infection, there is a shift within class Clostridia from dominant effects of Lachnospiraceae on immunity to greater impact of Veillonellaceae. Seven of 67 interactions were significantly correlated in both RhCMV− and RhCMV+ animals, but with opposite polarity (Table 2; Fig. 4A, bottom section; and Fig. 4 B–C). *Oribacterium*, *Roseburia*, and *Faecalibacterium* are all known short-chain fatty acid producers. As expected, these genera have negative correlations with cytokine-producing CD8^+^ T cells in RhCMV-seronegative animals; however, the opposite is true for seropositive animals (Fig. 4 B–C).

**Table 2:** Results of multiple linear regression resulting in significant interaction due to RhCMV infection

Bacterial genus	Immune marker	RhCMV-		RhCMV+		***P***_***interaction***_	Adjusted ***R***^**2**^	Significance in group
		***P***	Coef.	***P***	Coef.			
*Bacteroides*^*b*^	CD4 memory	0.0158	0.41	0.4790	−0.12	0.0265	0.21	RhCMV− only
	CD4 naïve	0.0169	−0.39	0.4661	0.12	0.0280	0.26	
*Bulleidia*^*a*^	CD25^+^, CD127^lo^, CD4	0.0263	0.28	0.4804	−0.18	0.0302	0.33	
*Catenibacterium*^*a*^	HLADR^+^, CD38^+^, CD8_mem_	0.0063	0.41	0.0690	−0.36	0.0025	0.23	
*Coprococcus*^*a*^	KI67^+^, CD8_mem_	0.0054	−0.35	0.1863	0.31	0.0076	0.21	
	KI67^+^, CD8_eff_	0.0011	−0.38	0.3944	0.14	0.0093	0.49	
	KI67^+^, CD4_eff_	0.0248	−0.35	0.2477	0.22	0.0244	0.18	
	TNF^+^, CD8_mem_	0.0116	−0.29	0.2993	0.23	0.0261	0.29	
	TNF^+^, CD4	0.0002	−0.52	0.9832	0.00	0.0290	0.25	
	CD4 naïve	0.0036	0.40	0.7718	−0.06	0.0482	0.29	
*Dialister*^*b*^	IL17^+^, CD4_mem_	0.0041	−0.53	0.1723	0.29	0.0005	0.22	
	IL17^+^, CD4	0.0168	−0.48	0.1954	0.29	0.0021	0.11	
*Dorea*^*a*^	KI67^+^, CD4_eff_	0.0215	−0.54	0.6508	0.06	0.0200	0.17	
	IFN^+^, CD8_mem_	0.0454	−0.42	0.3354	0.05	0.0494	0.28	
*Faecalibacterium*^*a*^	TNF^+^, CD8	0.0012	−0.41	0.0529	0.34	0.0006	0.37	
	IFN^+^, TNF^+^, CD8	0.0019	−0.38	0.0523	0.34	0.0008	0.36	
	IL17^+^, CD4	0.0123	−0.44	0.2114	0.29	0.0036	0.10	
	IFN^+^, CD8_eff_	0.0131	−0.43	0.1782	0.25	0.0038	0.21	
	TNF^+^, CD4	0.0063	−0.46	0.2805	0.21	0.0057	0.19	
	KI67^+^, CD4_eff_	0.0046	−0.50	0.6184	0.08	0.0142	0.21	
*Flexispira*	TNF^+^, CD4	0.0018	0.56	0.6954	0.07	0.0406	0.20	
	IFN^+^, CD4_mem_	0.0233	0.35	0.8025	−0.03	0.0490	0.48	
*Lachnospira*^*a*^	TNF^+^, CD8_mem_	0.0196	−0.29	0.1668	0.25	0.0155	0.30	
	TNF^+^, CD4	0.0391	−0.32	0.3635	0.18	0.0416	0.14	
*Oribacterium*^*a*^	IL17^+^, CD4	0.0255	−0.45	0.0767	0.37	0.0008	0.14	
	TNF^+^, CD4	0.0027	−0.52	0.4137	0.13	0.0053	0.20	
	KI67^+^, CD8	0.0063	−0.20	0.2347	0.15	0.0204	0.66	
	IFN^+^, CD4	0.0318	−0.43	0.6682	0.08	0.0212	0.25	
	CD4 memory	0.0304	−0.37	0.2192	0.15	0.0265	0.19	
	CD4 naïve	0.0239	0.36	0.2736	−0.12	0.0329	0.25	
	IFN^+^, TNF^+^, CD8_mem_	0.0448	−0.23	0.0639	0.22	0.0433	0.25	
*p-75-a5*	KI67^+^, CD8_mem_	0.0259	0.29	0.1945	−0.30	0.0217	0.18	
*Prevotella*^*b*^	KI67^+^, CD4_eff_	0.0070	−0.45	0.9735	0.00	0.0470	0.20	
*Roseburia*^*a*^	TNF^+^, CD8_eff_	0.0018	−0.39	0.0737	0.42	0.0024	0.29	
	IFN^+^, CD8	0.0231	−0.24	0.1001	0.40	0.0104	0.36	
	KI67^+^, CD4_eff_	0.0073	−0.39	0.1415	0.31	0.0126	0.21	
	IFN^+^, CD8_eff_	0.0148	−0.37	0.3144	0.26	0.0231	0.21	
	KI67^+^, CD8	0.0184	−0.14	0.2526	0.23	0.0396	0.66	
*Ruminococcus*^*a*^	B cells	0.0004	0.68	0.6608	−0.07	0.0038	0.15	
*Sutterella*	CD4 memory	0.0003	0.60	0.5867	0.07	0.0196	0.29	
	CD4 naïve	0.0002	−0.59	0.4672	−0.09	0.0234	0.35	
*YRC22*	CD4 memory	0.0094	0.50	0.3128	−0.14	0.0059	0.23	
	CD4 naïve	0.0108	−0.47	0.3415	0.13	0.0075	0.28	
	CD8 memory	0.0072	0.50	0.6365	−0.09	0.0163	0.12	
*[Prevotella]*	TNF^+^, CD8	0.1985	−0.18	0.0424	0.38	0.0131	0.30	RhCMV+ only
	TNF^+^, CD8_mem_	0.1972	−0.21	0.0401	0.32	0.0187	0.29	
*Anaerovibrio*^*b*^	KI67^+^, CD8	0.5872	0.03	0.0074	0.41	0.0111	0.70	
	CD8 memory	0.1324	0.26	0.0206	−0.38	0.0124	0.11	
	KI67^+^, CD4_eff_	0.7736	−0.05	0.0079	0.45	0.0470	0.15	
*Catenibacterium*^*a*^	B cells	0.8095	−0.04	0.0036	0.69	0.0073	0.13	
	KI67^+^, CD8_mem_	0.5884	−0.07	0.0077	−0.62	0.0303	0.22	
*Megasphaera*^*b*^	IFN^+^, CD8	0.9447	0.01	0.0166	0.44	0.0425	0.36	
*Oribacterium*^*a*^	TNF^+^, CD8	0.0720	−0.21	0.0331	0.33	0.0139	0.30	
*Prevotella*^*b*^	TNF^+^, CD8	0.3131	−0.12	0.0292	0.37	0.0220	0.30	
	TNF^+^, CD8_mem_	0.6065	−0.05	0.0061	0.39	0.0417	0.29	
*Roseburia*^*a*^	IFN^+^, TNF^+^, CD8_mem_	0.0519	−0.19	0.0238	0.48	0.0115	0.28	
*Streptococcus*	CD4	0.5786	0.04	0.0383	0.83	0.0097	0.32	
*Veillonella*^*b*^	CD4	0.0677	0.19	0.0183	−0.33	0.0144	0.30	
	TNF^+^, CD4	0.3040	−0.19	0.0418	0.32	0.0305	0.13	
	IFN^+^, CD8_eff_	0.3388	−0.19	0.0420	0.30	0.0364	0.15	
*Faecalibacterium*^*a*^	TNF^+^, CD8_mem_	0.0010	−0.44	0.0273	0.32	0.0004	0.37	Both RhCMV− and RhCMV+
	IFN^+^, TNF^+^, CD8_mem_	0.0085	−0.36	0.0264	0.33	0.0019	0.32	
	IFN^+^, CD8_mem_	0.0221	−0.36	0.0179	0.28	0.0035	0.33	
*Oribacterium*^*a*^	IFN^+^, CD8	0.0197	−0.29	0.0296	0.34	0.0023	0.38	
	TNF^+^, CD8_mem_	0.0299	−0.25	0.0471	0.23	0.0293	0.28	
*Roseburia*^*a*^	TNF^+^, CD8	0.0166	−0.24	0.0284	0.53	0.0028	0.34	
	TNF^+^, CD8_mem_	0.0311	−0.21	0.0222	0.47	0.0092	0.30	

^a^Gram-positive and ^b^gram-negative bacterial genera known to produce SCFA

## Discussion

We found that CMV infection is associated with alterations in both gut microbes and relationships between microbes and host immunity. A substantial literature demonstrates the effects of both human and rhesus cytomegaloviruses on host immune function [4, 9, 24, 29, 49, 50]. Brodin and colleagues showed that HCMV infection impacted more than 50% of the immune parameters examined [2]. Our data suggest the possibility that altered host sensitivity to microbial constituents forms part of the mechanism of these CMV effects—that is, CMV may act partly via changed sensitivity of the host to its own microbiota. Thus, understanding the effects of CMV on host immunity requires full understanding of interactions between chronic viral infections, microbes, and the immune system.

While previous studies did not find drastically changed host microbiota during RhCMV infection as assessed by beta diversity measures [24, 51, 52], we found significant differences between seronegative and seropositive microbiotas using methods for compositional data analysis [53], albeit with some overlap between the two groups (Fig. 1B). RhCMV-seropositive animals demonstrated increased abundance of *Streptococcus* and decreased abundances of *Blautia*, *Butyrivibrio*, and *Sarcina*—consistent with a previous report of decreased Firmicutes abundance in CMV-infected human infants [52]. We used different methods to identify differentially abundant individual taxa (limma-voom) vs. compositionally altered microbial communities (elastic net). Elastic-net regression identified an additional 15 genera that were associated with RhCMV seropositivity, while agreeing with many individual results from the limma-voom analysis, e.g., *Streptococcus* and *Butyrivibrio*. A separate longitudinal cohort of macaques administered RhCMV-vectored vaccines was used to test the machine-learning results; these experimentally infected macaques demonstrated similar changes in their microbiotas, indicating a causal relationship between RhCMV infection and microbiota changes.

All taxa that declined in abundance during RhCMV infection are SCFA-producing genera [44–46]. Changes in abundances of SCFA producers in the gut are commonly associated with altered diets, with higher fiber intake resulting in greater abundance of SCFA producers and increased host health [54, 55]. All animals in our study were fed the same diet, however, likely increasing sensitivity of the experiment to RhCMV-imposed changes. Previous research has demonstrated reduced abundance of SFCA producers in people suffering from inflammatory bowel disease, especially Crohn’s disease [56, 57], suggesting that reduced abundance of these taxa is a correlate of systemic inflammation. In a parallel line of experimentation, we demonstrated that RhCMV infection induces outgrowth of innate-memory CD8^+^ cells through an IL-15-dependent pathway [9]. While IL-15 may be important for protection against SIV, the cytokine has also been shown to promote intestinal dysbiosis, manifesting as reduction in SCFA producers [58], similar to the finding in this study (Fig. 2 A–B). We hypothesize that the changes in bacterial abundance reported above are due to altered host-gene expression, including IL-15 expression, in gut tissue that is caused by the immune response to RhCMV.

Interruptions of microbial influence on the host immune system due to RhCMV infection were observed mostly in T-cell subsets. Previously, the microbiome has been implicated in the development of mucosal Th17 cells and Tregs [59, 60]. Th17 cells and Tregs, present ubiquitously at the mucosal surface, are both induced by TGF-β signaling during activation. In the presence of IL-6, naïve CD4^+^ T cells commit to the Th17 lineage, while in the absence of IL-6 and other pro-inflammatory molecules, to the Treg lineage [61]. A study in mice found that supplementation with *Faecalibacterium* significantly decreased both IL-17A expression and levels of IL-17 in the plasma [62]. We similarly observed inverse correlations between abundances of *Faecalibacterium*, *Dialister*, and *Oribacterium* and the frequency of circulating Th17 cells among RhCMV-seronegative (but not -seropositive) animals. Another SCFA-producing genus, *Bulleidia* [63], positively correlated with Tregs in RhCMV-seronegative animals but not RhCMV-seropositive animals (Fig. 4 A–B). RhCMV infection therefore disrupts the relationship between SCFA producers and Th17/Treg balance, supplanting a homeostatic mechanism that controls this balance in seronegative macaques.

While in this example RhCMV infection subverts a preexisting gut microbe-immune system relationship, in other instances, RhCMV appears to sensitize its host to microbial influence: *Anaerovibrio*, *Megasphaera*, and *Veillonella* are important correlates of certain immunophenotypes only in RhCMV-seropositive macaques. While previously classified in the Clostridiales order based on 16S rRNA gene sequences and metabolic characteristics (all are SCFA producers) [46], these genera are gram negative in contrast to other members of Clostridiales and have been reclassified to a novel bacterial order, Negativicutes [64]. Among RhCMV-seropositive macaques, *Anaerovibrio* positively correlates with T-cell proliferation, while *Megasphaera* and *Veillonella* positively correlate with T-cell effector functions. Lipopolysaccharide (LPS), a key component in the cell wall of gram-negative bacteria, stimulates T cells via engagement of toll-like receptor 4 (TLR4) within innate immune cells [65, 66]. In addition, studies have shown that LPS promotes immediate-early gene expression of HCMV [67] and reactivation of latent CMV in mice [68]. Thus, CMV and the altered microbiota may synergistically promote T-cell activation, which in turn promotes further CMV replication.

Surprisingly, RhCMV infection inverts the relationship between abundance of SCFA producers and effector CD8^+^ T cells that is seen in RhCMV-naïve macaques. *Oribacterium*, *Roseburia*, and *Faecalibacterium* negatively correlate with the frequency of effector CD8^+^ T cells in seronegative macaques but positively correlate in RhCMV-seropositive animals (Fig. 4A). SCFA are thought to reduce inflammation via blockade of NF-κB activation [69–72] and inhibition of histone deacetylase [73–77]. However, SCFA can also cause inflammation when other TLR agonists are present [78] and cause inflammation when interacting with different G protein-coupled receptors [79, 80], suggesting that the net effect of SCFA partly depends on microenvironmental factors. In fact, SCFA has been shown to enhance the susceptibility to and induce the replication of CMV in human cell lines [81–84]. Therefore, CMV infection may compromise the anti-inflammatory signaling normally generated by the host’s endogenous microbiota and harness the SCFA produced by these bacteria to enhance its own survival.

In summary, we found that RhCMV infection was associated with a profound change in the relationship of an infected host to its microbiota. The methods we employed likely limited sensitivity of our study to other examples of altered host-microbe relationships, as 16S rRNA sequencing surveys bacterial taxonomy but not bacterial functions. A future study using metagenomic techniques would better resolve functions between bacteria and would possibly reveal more bacteria-immune system relationships that were impacted by RhCMV. Bacteria within a genus, or even within a species, maybe have different functions and consequently different effects on the host [85]. Additionally, gut-specific immune and transcriptomic data would provide mechanistic insights into the local interactions occurring between microbiome and host during RhCMV infection. Previous studies have shown that depleting Tregs in the salivary gland but not in the spleen resulted in CMV reactivation at one site but not the other [86], so profiling the gut immune system would likely reveal other interactions with CMV that are not seen in circulating cells. Nevertheless, our work provides a framework for discovering interactions between chronic viral infections and gut commensal microorganisms *in vivo*. Our future studies will use this framework to understand the importance of RhCMV for adaptive immune responses to infection or vaccination.

## Conclusions

RhCMV infection is associated with a profound change in the relationship of an infected host to its microbiota. SCFA-producing genera are found in lower abundance in RhCMV-infected macaques. Furthermore, relationships between gut bacteria and host immune functions are disrupted. We observed inverse correlations between abundances of SFCA producers and the frequency of circulating Th17 cells among RhCMV-seronegative—but not -seropositive—animals. Another SCFA-producing genus, *Bulleidia* [63], positively correlated with Tregs in RhCMV-seronegative but not RhCMV-seropositive animals. In addition, RhCMV infection inverts the relationship between abundance of SCFA producers and effector CD8^+^ T cells that is seen in RhCMV-uninfected macaques. *Oribacterium*, *Roseburia*, and *Faecalibacterium* negatively correlated with the frequency of effector CD8^+^ T cells in seronegative macaques but positively correlate in RhCMV-seropositive animals. RhCMV infection thus compromises the anti-inflammatory signaling normally generated by the host’s endogenous microbiota and harnesses the SCFA produced by these bacteria to enhance its own survival. Thus, part of the mechanism of vast CMV effects on host immunity is alteration of the host-microbiome relationship.

## Supplementary Information

**Additional file 1: Table S1.** Spearman correlations between immune cell subsets and CMV-microbe score.

## Data Availability

The datasets generated and analyzed during the current study are available in the SRA under BioProject ID PRJNA789338 and GitHub (https://github.com/HOC-Lab/cur01).
