# Supplementary material for: Cytomegalovirus infection disrupts the influence of short-chain fatty acid producers on Treg/Th17 balance
Source: Microbiome. 2022 Oct 10;10:168. doi: 10.1186/s40168-022-01355-3 (PMC9549678; doi:10.1186/s40168-022-01355-3)
Supplement: Supplementary file 2 — Additional file 1: Table S1. Spearman correlations between immune cell subsets and CMV-microbe score. [file 40168_2022_1355_MOESM1_ESM.docx]

Table S1. Spearman correlations between immune cell subsets and CMV-microbe score.

| **Immune cell subset** | **rho** | ***P* value** | **Adjusted *P* value** |
| --- | --- | --- | --- |
| IL4^+^, CD4_mem_ | 0.73 | 2.00E-12 | 1.66E-10 |
| IL4^+^, CD4 | 0.72 | 4.94E-12 | 2.05E-10 |
| IL4^+^, CD4_eff_ | 0.68 | 1.89E-10 | 5.23E-09 |
| IL4^+^, CD8_mem_ | 0.65 | 1.99E-09 | 4.13E-08 |
| CD8 naïve | -0.63 | 1.12E-08 | 1.86E-07 |
| CD8 effector | 0.63 | 1.46E-08 | 2.02E-07 |
| IL4^+^, CD8 | 0.61 | 4.12E-08 | 4.88E-07 |
| KI67^+^, CD8 | 0.60 | 8.93E-08 | 9.26E-07 |
| CD4 effector | 0.59 | 1.29E-07 | 1.19E-06 |
| IL4^+^, CD8_eff_ | 0.57 | 4.41E-07 | 3.66E-06 |
| IFN^+^, CD4_mem_ | 0.57 | 5.89E-07 | 4.44E-06 |
| TNF^+^, CD4_mem_ | 0.56 | 8.06E-07 | 5.57E-06 |
| IFN^+^TNF^+^, CD4_mem_ | 0.54 | 2.33E-06 | 1.49E-05 |
| KI67^+^, CD8_eff_ | 0.54 | 3.92E-06 | 2.33E-05 |
| IFN^+^, CD4_eff_ | 0.50 | 1.87E-05 | 1.03E-04 |
| IFN^+^TNF^+^, CD4_eff_ | 0.50 | 1.98E-05 | 1.03E-04 |
| IFN^+^, CD8_mem_ | 0.48 | 3.88E-05 | 1.89E-04 |
| CD83^+^, monocytes | -0.47 | 5.96E-05 | 2.75E-04 |
| IFN^+^, CD8 | 0.47 | 6.45E-05 | 2.82E-04 |
| CD8 | 0.46 | 8.77E-05 | 3.64E-04 |
| CD80^+^, B cells | -0.45 | 1.21E-04 | 4.79E-04 |
| TNF^+^, CD4_eff_ | 0.45 | 1.28E-04 | 4.83E-04 |
| CD56^−^CD16^+^, NK cells | 0.45 | 1.56E-04 | 5.62E-04 |
| CD4 | -0.44 | 1.79E-04 | 6.19E-04 |
| CD56^+^CD16^−^, NK cells | -0.43 | 2.89E-04 | 9.58E-04 |
| CD86^+^, monocytes | -0.43 | 3.04E-04 | 9.70E-04 |
| IFN^+^TNF^+^, CD8 | 0.42 | 4.34E-04 | 1.33E-03 |
| IFN^+^TNF^+^, CD8_mem_ | 0.41 | 5.28E-04 | 1.57E-03 |
| IL17^+^, CD8_eff_ | 0.41 | 5.79E-04 | 1.66E-03 |
| CD83^+^, B cells | -0.41 | 6.10E-04 | 1.69E-03 |
| TNF^+^, CD8 | 0.40 | 9.32E-04 | 2.47E-03 |
| TNF^+^, CD8_mem_ | 0.39 | 9.52E-04 | 2.47E-03 |
| IFN^+^TNF^+^, CD8_eff_ | 0.39 | 1.02E-03 | 2.51E-03 |
| CD86^+^, B cells | -0.39 | 1.03E-03 | 2.51E-03 |
| IL17^+^, CD4_mem_ | 0.39 | 1.11E-03 | 2.63E-03 |
| TNF^+^, CD8_eff_ | 0.38 | 1.43E-03 | 3.29E-03 |
| KI67^+^, CD4 | 0.38 | 1.50E-03 | 3.38E-03 |
| NK-CTL | 0.37 | 1.77E-03 | 3.87E-03 |
| IFN^+^, CD8_eff_ | 0.37 | 1.94E-03 | 4.14E-03 |
| IFN^+^TNF^+^, CD4 | 0.37 | 2.09E-03 | 4.34E-03 |
| CD4 naïve | -0.35 | 3.37E-03 | 6.83E-03 |
| IFN^+^, CD4 | 0.34 | 4.69E-03 | 9.26E-03 |
| CD25^+^CD127^lo^, CD4 | 0.34 | 4.95E-03 | 9.55E-03 |
| NK cells | 0.33 | 5.76E-03 | 0.01 |
| IL17^+^, CD8_mem_ | 0.32 | 8.78E-03 | 0.02 |
| CD80^+^, monocytes | -0.31 | 0.01 | 0.02 |
| Monocytes | 0.31 | 0.01 | 0.02 |
| CD4 memory | 0.29 | 0.02 | 0.03 |
| IL17^+^, CD8 | 0.27 | 0.03 | 0.04 |
